# An Association of Pathogens and Biofilms with Alzheimer’s Disease

**DOI:** 10.3390/microorganisms10010056

**Published:** 2021-12-28

**Authors:** Sandhya T. Chakravarthi, Suresh G. Joshi

**Affiliations:** 1 Center for Surgical Infection and Biofilm, College of Medicine, Drexel University, Philadelphia, PA 19104, USA; tara.chakravarthi@gmail.com; 2 Drexel School of Biomedical Engineering, Science & Health Systems, Drexel University, Philadelphia, PA 19104, USA

**Keywords:** Alzheimer’s disease, beta-amyloid, *Borrelia*, dementia, infectious agent, microbial biofilm, neuronal death, pathogen, spirochete, *Treponema*

## Abstract

As one of the leading causes of dementia, Alzheimer’s disease (AD) is a condition in which individuals experience progressive cognitive decline. Although it is known that beta-amyloid (Aβ) deposits and neurofibrillary tangles (NFT) of tau fibrils are hallmark characteristics of AD, the exact causes of these pathologies are still mostly unknown. Evidence that infectious diseases may cause AD pathology has been accumulating for decades. The association between microbial pathogens and AD is widely studied, and there are noticeable correlations between some bacterial species and AD pathologies, especially spirochetes and some of the oral microbes. *Borrelia burgdorferi* has been seen to correlate with Aβ plaques and NFTs in infected cells. Because of the evidence of spirochetes in AD patients, *Treponema pallidum* and other oral treponemes are speculated to be a potential cause of AD. *T. pallidum* has been seen to form aggregates in the brain when the disease disseminates to the brain that closely resemble the Aβ plaques of AD patients. This review examines the evidence as to whether pathogens could be the cause of AD and its pathology. It offers novel speculations that treponemes may be able to induce or correlate with Alzheimer’s disease.

## 1. Introduction

Alzheimer’s disease (AD) is a form of dementia that causes the decline of multiple cognitive areas, such as personality and behavior, language, motor skills, and memory [1,2]. It is currently the most prevalent cause of dementia worldwide [2]. The pathology of AD is described as neurofibrillary tangles and amyloid plaques. Beta-amyloid deposits (Aβ) accumulate during the processing of β-amyloid precursor protein (AβPP) [3]. Usually, Aβ is degraded, but in AD, it accumulates in plaques between nerve cells. Neurofibrillary tangles are formed when tau is misfolded during the formation of neuron microtubules. Clinically, it is thought that these Aβ and neurofibrillary tangles lead to the symptoms of AD [1,2]. However, it is not entirely understood why these pathologies occur, what causes amyloid plaques, and why tau becomes misfolded.

Because the exact cause of AD remains unclear, scientists have been trying to determine if pathogens could contribute to the development of AD. There is evidence of various fungi, viruses, parasites, and bacteria being prevalent in AD patients’ brains, and some have an association with AD pathology. Aβ is an antimicrobial peptide, so it is speculated that individual organisms invade the brain and lead to a buildup of Aβ. Microtubulin, polysaccharides, and DNA of the fungi *Candida albicans* have been detected in the neurons of AD patients, indicating that the fungi can disseminate into the brain during infection, and potentially cause the aggregation of Aβ and lead to plaque formation [4,5,6]. The parasitic organism *Toxoplasma gondii* is an intracellular pathogen, and it has been seen that *T. gondii* can co-localize with Aβ plaques in mouse brains [7]. Hyperphosphorylated tau can appear in just 15 days following infection, suggesting that *T. gondii* might lead to both hallmark characteristics of AD [7].

It is known that infections with viruses can lead to other diseases, such as in human papillomavirus and its association with cervical cancer. Therefore, there is reason to believe that viruses could induce other conditions, such as AD. Herpes simplex virus type I is one of the most studied viruses as a potential cause of AD. Upon progression, HSV-1 can lead to herpes simplex encephalitis, affecting the brain’s frontal and temporal lobes, leading to similar clinical symptoms seen in AD patients [8]. Viral DNA has been observed in the brains of AD patients, and the DNA can be detected in Aβ plaques [8,9]. Additionally, HIV-1 is associated with abnormal phosphorylation of tau, another hallmark of AD [10]. Human cytomegalovirus (CMV) infection has also been observed to correlate with AD pathology. Because CMV infection causes the release of IFN-γ and TNF-α, both associated with Aβ plaques, it has been speculated that the virus could lead to AD [11]. In some human cells, Aβ plaques can be induced by CMV [11].

Bacteria seem to be the most studied pathogens when looking for pathogenic causes of AD. Several studies have provided evidence that *Chlamydiophila pneumoniae*, a bacterium that causes respiratory disease, is found in AD brain tissue samples based on PCR analysis, and in some cases, *C. pneumoniae* is culturable from these brains [12,13]. Also, *C. pneumoniae* infects the primary cells affected by AD, microglia, which are observed to co-localize with Aβ plaques [14]. Cells infected with *C. pneumoniae* can also co-localize with NFTs in AD brains [13]. However, there is conflicting evidence that *Chlamydiophila pneumoniae* is present in AD brain samples, as similar studies failed to reproduce the previously described observations [15,16]. Nevertheless, there is evidence that the bacterium could have an association with AD pathology.

A pathogen’s ability to disseminate to the brain and infect neurons is critical if it can cause AD. The causative agent of Rocky Mountain spotted fever (RMSF), *Rickettsia rickettsii*, has been seen to infect neurons and lead to cell death via apoptosis, which is the fate of neurons in AD [17,18]. A pathogen related to *R. rickettsii*, *Coxiella burnettii*, the bacterium that causes Q fever, can have neurological involvement [19]. However, although they are considered when speculating a pathogenic cause of AD, there is little evidence to suggest a correlation.

*Helicobacter pylori* has been of great interest recently, as it was discovered to contribute to the development of gastric ulcers and cancer, as well as extra-gastric diseases [20,21]. Studies have shown that *H. pylori*-specific antibodies are present in the cerebrospinal fluid of AD patients, and mice infected with the bacterium demonstrate impaired cognitive abilities [21,22]. Also, infected rats had increased Aβ levels, causing memory impairment [22]. Tau hyperphosphorylation can also be observed in hippocampal neural fibers of mice infected with *H. pylori*, and hyperphosphorylation of tau increased in neuroblastoma cells incubated with the bacterium [23]. Therefore, there is reason to speculate about the association of *H. pylori* and AD.

Another group of bacteria of interest when examining the correlations between bacteria and AD is periodontal bacteria. These bacteria cause periodontal disease, often in elderly individuals, as cognitive decline increases [24]. If these bacteria can disseminate to the brain, they could cause inflammation and lead to AD pathology [25]. *Porphyromonas gingivalis*, which becomes increasingly prevalent in the oral microbiota as individuals age, can become pathogenic and contribute to periodontal disease [26]. This bacterium releases specific proteases that stimulate an immune response, causing inflammation [26]. These proteases, called gingipains, have been observed in AD brains and may be associated with Aβ plaques and tau load [26]. Because *P. gingivalis* can disseminate to the brain, there is reason to believe that AD pathology could be induced by this bacterium [26].

Spirochetes are some of the most in-depth studied organisms when looking for a pathogenic cause of AD. Early evidence has shown that spirochetes are present in most AD patients studied and can be visualized in AD brains in association with Aβ plaques [27]. Based on this early evidence and the observations that some spirochetes cause neurological symptoms, several spirochetes were proposed to have the most likely association with Alzheimer’s Disease. One of these is the causative agent of Lyme disease, *Borrelia burgdorferi*, a zoonosis transmitted by ticks of the genus *Ixodes*. Generally, Lyme disease causes a skin lesion upon initial infection, and if left untreated, the infection can spread to other organs [28]. When Lyme disease progresses to neurological involvement, patients can develop meningitis, encephalitis, and myelitis [28]. It is encephalomyelitis that can lead to symptoms similar to those seen in AD patients, including visual disturbances, speech impairments, and dementia [28]. *B. burgdorferi* DNA is detectable in AD brains, and Aβ plaque aggregation is observed in brain tissues infected with the bacterium [27,29]. Additionally, hyperphosphorylation of tau can be seen in infected microglia, suggesting that *B. burgdorferi* could be a potential cause of AD and its pathology [30].

Another spirochete of interest is *Treponema pallidum*, the bacteria that causes syphilis. Syphilis progresses in three stages: primary, secondary, and tertiary syphilis. Primary syphilis is localized to the genitals and is characterized by a chancre. If untreated, the disease progresses to secondary syphilis when the infection spreads throughout the body. Again, if no treatment is given, the disease will progress to tertiary syphilis, wherein many organs can be damaged. Among them, the brain may be affected in neurosyphilis, when syphilis affects the brain and spinal cord [29]. While there are several neurosyphilis types, general paresis clinically presents with symptoms similar to AD, such as dementia, personality changes, and general cognitive impairments [29]. Earlier evidences of *T. pallidum* in the brain showed that the bacterium could form neurofibrillary tangles, and Aβ aggregates closely resemble those seen in AD brains [31].

With evidence that treponemes are present in the periodontal environment, and spirochetes can disseminate to the brain, oral treponemes may be a pathogenic cause of AD. This review will examine and summarize the evidence that there is a pathogenic cause of AD. The fungi, parasites, viruses, and bacteria that have been shown to correlate with AD pathology will be highlighted. Specifically, spirochetes will be scrutinized in depth. The purpose of this review is to determine if there is a spirochete that is the most likely cause of AD and to justify why it should be explored further. The goal is to provide support and substantiate a reason for future research of a specific spirochete.

This review is written, based on the research that was conducted fully through literature searches using online databases. PubMed Central was almost exclusively used, in addition to general science citation in Google search. Keywords for preliminary searches included: Alzheimer’s Disease, bacteria, beta-amyloid, biofilm, dementia, microbial pathogens, neurofibrillary tangles, spirochete; tau, virus, etc. in permutation combinations. As more evidence was gathered for individual organisms, the keywords used for literature searches were more specific to the organism or disease that was in question. For example, Borrelia; Lyme disease; Treponema; periodontal disease; Herpes Simplex Virus; etc. The citation software used for insertion and formatting of citations was EndNote X7.

## 2. Microbes as Potential Cause of AD

There is increasing evidence that certain pathogens may be involved in the development of AD, known as the AD pathogen hypothesis [32]. Bacteria, viruses, and fungi have all been investigated as having a role in AD. This stems from the observation that Aβ acts as an antimicrobial peptide in the innate immune response. Therefore, Aβ production may be triggered by a pathogen invading the brain and CNS, leading to the formation of plaques seen in AD patients. Table 1 shows the microbial association with AD.

It is hypothesized that fungal infections may contribute to the development of AD. Fungal proteins can be detected in brain tissue samples from AD patients, and some fungi, such as *Candida albicans*, are susceptible to killing by Aβ [5]. Pisa et al. used immunofluorescence staining with anti-*Candida glabrata* antibodies and anti-tubulin to observe the presence of fungal material in human brain tissue from the frontal cortex of AD brains and normal brains [5]. Fungal microtubules were present in the cytoplasm of neurons, although the fungal material’s quantity and frequency varied between AD patients’ brains [5]. Using *C. glabrata*-specific antibodies, it was shown that the fungal cells are present in about 10% of neurons isolated from the brains of patients diagnosed with AD before death [5]. Components of other fungal species, such as *C. albicans* and *Penicillium notatum,* can also be detected in AD brain tissue, indicating that mixed fungal infections may play a role in AD development [5]. Additionally, polysaccharides and DNA of fungi have been detected in brain tissue samples from AD patients, demonstrating that fungal infections may disseminate in AD patients and contribute to AD progression [4]. In addition to AD patients’ frontal cortexes, fungal components are detectable in the cerebellar hemisphere, hippocampus, and choroid plexus in diseased brains [6]. The neurons of some brain samples contained whole fungal cells [6]. Tau can also be seen in higher quantities in neurons that also had fungal structures [6]. Given that NFTs of tau are a hallmark of AD, these findings suggest that fungi in brain tissue can increase tau, potentially contributing to AD progression. Fungi may also be correlated with the presence of Aβ plaques. These plaques are frequently observed in the blood vessels of AD patients. Pisa et al. observed fungal hyphae in AD patients’ capillaries, indicating that fungi can infect AD patients’ blood vessels and may contribute to Aβ plaque growth [6]. Taken together, the observations that various species of fungi are present in AD brain tissue, there is increased tau in fungi-infected neurons, and fungal components are detectable in AD patients’ blood vessels suggest that fungal infections could potentially be a contributing factor in AD.

Evidence has been accumulating that *Toxoplasma gondii* infection may induce some of the hallmark characteristics seen in AD brains. *T. gondii* is an intracellular parasite, and when the cyst form of the protozoa is ingested, it replicates in the gut and can travel throughout the body and form cysts in the CNS and brain [7]. These cysts can persist in the brain for the remainder of an infected individual’s life, and infections can often be asymptomatic in immunocompetent humans, acting as an intermediate host. It is estimated that more than one billion people are infected worldwide, although prevalence varies by geographical region [7,33]. Because seroprevalence increases with age, it is thought that infection with *T. gondii* could be associated with AD in aging populations. Mice infected with *T. gondii* show Aβ plaque formation, especially co-localized to the *T. gondii* cysts in mouse brains [7]. Due to Aβ plaques being a hallmark of AD, these results suggest that *T. gondii* may induce these plaques. It is also seen that the parasite can cause hyperphosphorylation of tau [7]. Infected mice began showing hyperphosphorylated tau 15 days post infection, and the presence of pTau continued increasing as time after infection increased [7]. Collectively, these findings suggest that infection with *T. gondii* cysts can induce both hallmark characteristics of AD and potentially lead to the disease’s development.

In addition to inducing the hallmarks of AD, it is thought that *T. gondii* can affect neuronal cells, leading to the alteration of the neurotransmitter glutamate controlled by the N-methyl-D-aspartate receptor (NMDAR) [7]. The NMDAR is a receptor on neuronal cells that is involved in synaptic transmission, and it has been seen that the presence of Aβ can impair the function of NMDAR [7]. Research has shown that NMDAR function is disrupted in AD, possibly causing some of the symptoms of the disease [7]. When mice were infected with *T. gondii*, NMDAR expression decreased in the cortex and hippocampus as post-infection time increased, and the neurons experiencing the loss were co-localized with *T. gondii* cysts [7]. These results suggest that NMDARs can be lost when *T. gondii* is present. Additionally, one of the symptoms of AD is the loss of olfaction. Torres et al. observed that *T. gondii* infection was correlated with a loss of VGLUT2, a glutamate transporter involved in transmitting signals between the nose and brain in sensory neurons [7]. Together, these findings indicate that *T. gondii* infection may lead to some of the pathophysiology seen in AD.

Of the viruses thought to be associated with AD, herpes simplex virus type 1 (HSV-1) is one of the most studied. Once infected with HSV-1, an individual carries the virus for the remainder of life in a latent form in the peripheral nervous system [8,9]. However, viral proteins are not present in latent infections, and only viral DNA can be found [9]. In elderly brains, HSV-1 was highly prevalent in patients with sporadic AD and patients without AD [8]. Because of this observation, the correlation between HSV-1 and the onset of sporadic AD remains unclear. HSV-1 can progress to herpes simplex encephalitis, leading to cognitive decline, impaired memory, and alterations in behavior, all of which are also seen in AD patients [8]. Additionally, both diseases affect the frontal and temporal lobes of the brain, which could explain the similarity of symptoms [8]. Since HSV-1 is prevalent in the brain tissue of both people with AD and those without, there might be a reason for why some HSV-1 patients develop AD and others do not. One possible risk factor of the development of AD in individuals infected with HSV-1 is a variant of apolipoprotein E gene (APOE-ε4) [34]. Because the majority of elderly AD patients infected with HSV-1 also possessed the APOE-ε4 gene, it is suspected that this gene is involved with AD progression [34].

Given that AD’s hallmark characteristics are extracellular Aβ plaques in brain tissue, Wozniak et al. hypothesized that amyloid plaques and HSV-1 DNA might closely reside together in brain tissue [9]. It was found that HSV-1 DNA and Aβ plaques were co-localized in the brains of elderly patients with AD [9]. After measuring the quantity of plaques in both the brains of AD patients and elderly brains without AD, it was seen that both populations had high numbers of plaques, and HSV-1 was detectable in the majority of plaques in both groups [9]. Due to these results, infection by HSV-1 may lead to Aβ plaque formation in general, and not just in AD patients [9].

Another hallmark characteristic of AD is neurofibrillary tangles (NFTs) of tau that are abnormally phosphorylated [10]. Tau can potentially be phosphorylated on specific locations, including serine–proline and threonine–proline motifs [10]. Due to the evidence that HSV-1 is correlated with the formation of Aβ plaques in AD patients, it is possible that the virus also contributes to the abnormal phosphorylation of tau seen in patients with AD [10]. Wozniak et al. illustrated that HSV-1 is associated with tau phosphorylation at positions T212 and S214, both of which are known tau phosphorylation sites of AD patients [10]. Additionally, it was seen that when cells were infected with HSV-1, abnormal phosphorylation of tau increased [10]. Taken together, the correlations demonstrated with HSV-1, Aβ plaque formation, and NFT development suggest that HSV-1 could be a potential cause of AD.

Human cytomegalovirus (CMV) causes often asymptomatic infection in most individuals, although it can cause mild to severe infections in immunocompromised people and elderly adults [11,35]. Viral seroprevalence increases among aging populations, and based on the examination of AD brains, there is evidence that CMV is associated with an AD diagnosis [11]. CMV is usually acquired during childhood and can remain latent for long periods, with reactivation possible later in life if an infected individual becomes immunocompromised from other diseases or aging. If CMV reactivates, virus-specific CD4+ and CD8+ T cells can increase, along with a decrease in CD27 and CD28 and increased CD57 [11]. This is known to lead to the secretion of IFN-γ and TNF-α by senescent cells, which is associated with the formation of Aβ plaques in AD patients [11]. Therefore, potential inflammation caused by CMV infection in the CNS may contribute to the development of AD, as there is growing evidence that inflammation is a contributing factor to AD onset and progression. Barnes et al. demonstrated the immunological features of seropositive individuals for CMV, who had been diagnosed with AD before death [11]. It was seen that the majority of CMV seropositive samples had increased IFN-γ levels in CSF, which correlated heavily with NFT density and minimally with AD diagnosis, and no seronegative samples had detectable IFN-γ [11]. Additionally, Aβ plaque formation seemed to be induced by CMV in human foreskin fibroblasts, indicating that the virus may be capable of causing the plaque formation seen in the neurons of AD patients [11]. Westman et al. observed a similar correlation between IFN-γ levels and AD diagnosis [36]. In the CMV seropositive experimental group, higher levels of IFN-γ were seen among AD individuals [36]. The results from these studies indicate that there may be an association between AD and human cytomegalovirus based on or due to IFN-γ production.

### 2.1. Bacteria as Potential Causative Agents of AD

#### 2.1.1. Chlamydia

Among several bacterial species that have shown an association with AD, *Chlamydiophila pneumoniae*, formerly *Chlamydia pneumoniae*, may have a role in the onset of the disease. *C. pneumoniae* is a Gram-negative bacterium that colonizes the respiratory tract and can cause pneumonia, bronchitis, and mild symptoms of a sore throat and cough [37]. Several studies have indicated that *C. pneumoniae* can be found in AD brains, although there is conflicting evidence. As AD is seen in aging populations, it was proposed that *C. pneumoniae* might have a link with the disease because antibodies to the bacterium increase in older individuals [12]. Balin et al. used polymerase chain reaction analysis to determine if *C. pneumoniae* is present in AD brain tissue from the hippocampus and temporal lobes of 19 AD patients [12]. It was seen that 17/19 AD brains sampled were positive for *C. pneumoniae*, whereas only one (1/37) patient without AD was positive [12]. Similar results were seen later by Gerard et al., where PCR was used to determine if *C. pneumoniae* DNA was present in AD and non-AD brain tissue [13]. In this study, 80% of AD brain tissue samples and 11.1% of non-AD brain tissue samples showed positive results for *C. pneumoniae* DNA [13]. Because the APOE-ε4 allele was previously associated with AD onset, the AD and non-AD patients were tested for the allele, and it was observed that 11/19 AD patients and 4/19 non-AD patients were positive for the allele [12]. These results were mirrored in a subsequent study that found 14/25 AD patients possessed the APOE-ε4 allele, while only 4/27 non-AD patients had the allele [13]. Therefore, it is suggested that *C. pneumoniae* presence could be correlated with late-onset AD development, and AD patients that possess the APOE-ε4 allele may be more at risk. Additionally, *C. pneumoniae* present in AD brain tissue was culturable [12]. Gerard et al. repeated the experiment on one AD sample that was highly positive for *C. pneumoniae* and observed that the bacterium was indeed culturable, indicating that the bacterium was viable and capable of causing active infection in patients [13]. There is also evidence that *C. pneumoniae* can enter the brain through the olfactory bulb, as it is a pathogen that affects the respiratory system, and this observation is indicated by findings of *C. pneumoniae* genetic material in the olfactory bulbs of AD patients [14]. Since AD is known to affect the olfactory bulb and cause a loss of the sense of smell, there may be a correlation between the bacterium and AD pathology wherein *C. pneumoniae* could cause the initial damage seen in the olfactory bulb [14]. Given that a hallmark characteristic of AD is the deposition of Aβ plaques in the brain, it is speculated that *C. pneumoniae* may contribute to the formation of these plaques. The release of pro-inflammatory cytokines and the high presence of monocytes and macrophages in response to *C. pneumoniae* infection could be associated with AD pathology, such as Aβ plaques [14]. Aβ plaques are known to cause inflammation in the brains of AD patients. Microglia, the cell type involved with inflammation in AD brains, have been seen to co-localize with Aβ plaques [12]. Microglia are also one of the dominant cell types that *C. pneumoniae* infects in the brain, as it is an intracellular pathogen [12]. Therefore, there seems to be a correlation between the inflammation caused by *C. pneumoniae*-infected microglia and the abnormal pathology observed in AD brains [14]. The other hallmark pathological feature of AD is NFTs. When brain tissue was examined for the location of *C. pneumoniae*-infected cells, it was found that they were co-localized with NFTs in AD brains [13]. Because Aβ plaques and NFTs are two of the hallmarks often used to diagnose AD, there may be a correlation of *C. pneumoniae*-infected individuals being at higher risk for developing AD.

Despite the inferences that can be elicited from the previously described studies, there is some conflicting evidence that *C. pneumoniae* may contribute to AD progression. In a study that analyzed brain tissue from 20 AD patients’ hippocampi, PCR was unsuccessful in detecting *C. pneumoniae* DNA, and immunocytochemistry methods did not reveal antigens to *C. pneumoniae* [15]. Similar results were seen in a separate study that examined brain tissue samples from six brain regions, including the hippocampus, frontal cortex, and parietal cortex, of 73 patients with AD and 28 patients without AD [16]. Upon PCR analysis, only three samples were positive in at least one region, but the positive results were not reproducible in subsequent experiments [16]. The researchers also attempted to culture *C. pneumoniae* from the tissue samples, and DNA was not detectable in the cultures [16]. Notably, a study that used tissue samples from the brains of the same AD patients examined by Balin et al. had conflicting results compared to the original study [12,38]. PCR did not reveal any positive results for *C. pneumoniae* DNA in the brain tissue samples from AD patients, nor did immunohistochemical analysis yield positive results for *C. pneumoniae* antibodies [38]. It should be mentioned that the majority of these studies, both those with the presence or absence of *C. pneumoniae* in AD brains, had relatively small sample sizes, ranging from 19–73 [12,13,14,15,38]. For subsequent studies, larger sample sizes may be necessary for a stronger correlation to be drawn. However, the limited availability of AD brains for research makes this challenging. Although the studies revealing the presence of *C. pneumoniae* in AD patients’ brain tissue indicated a possible cause of sporadic AD, the conflicting evidence from subsequent studies finding an absence of the bacteria suggests that additional studies are necessary before a definite relationship between *C. pneumoniae* and AD can be drawn.

#### 2.1.2. Rickettsia

RMSF is a bacterial infection that is transmitted by ticks, in which ticks transfer the causative agent, *Rickettsia rickettsii*, to the mammalian host. The gram-negative bacillus then infect endothelial cells, causing vascular symptoms [17]. These include rash, fever, and vasculitis, and the infection can progress to other organs if left untreated [17]. Among the organ systems affected, *R. rickettsii* can infect the central nervous system [39]. *R. rickettsii* is an obligate intracellular bacterium, and it is known to infect the endothelial cells of the host’s blood vessels, which contributes to the pathology of RMSF [40,41,42,43]. However, nervous system involvement is also seen as the disease progresses, leading to meningoencephalitis, seizures, confusion, delirium, vision loss, and facial nerve palsy [17,39]. Joshi and Kovacs for the first time demonstrated that *R. rickettsii* could infect the cerebellar granular neurons of rats, effectively leading to neuronal death via apoptosis, and neurons were more easily killed than endothelial cells infected with *R. rickettsii* [17]. Although the hallmark characteristics of AD are NFTs and Aβ plaques, the fate of neurons of AD brains is death by apoptosis [18]. It is possible that *R. rickettsii* can infect neurons and lead to the neuronal death seen in the later stages of AD. Several other Rickettsia species, including *Rickettsia akari*, *Rickettsia slovaca*, and *Rickettsia helvetica,* have also been shown to infect neurons and decrease the viability of the cells based on levels of intracellular ATP [44]. However, there is little evidence regarding *R. rickettsii* and AD’s pathology, so further studies are necessary to determine if there is a correlation between *R. rickettsii* infection and NFTs or Aβ plaques. Based on the observations that some *Rickettsia* species can lead to neuronal death, it is plausible that *R. rickettsii* may also be able to induce neuron death in AD patients.

#### 2.1.3. Coxiella

Another bacterium of interest is *Coxiella burnettii*, the intracellular bacterium that causes Q fever. Q fever is a zoonotic disease, as the bacterium resides in livestock and ticks [45]. Based on phylogenetic analysis, *C. burnettii* is classified as *Proteobacteria,* and it is related to the *Rickettsiella* genera [45]. When passed from a reservoir animal, humans inhale the aerosolized bacteria and become infected. Humans that develop acute illness can experience fever, flu-like symptoms, and pneumonia, but some humans remain asymptomatic [19,45]. As an intracellular bacterium, it has only been seen that *C. burnettii* infects macrophages and monocytes [45]. However, because it is related to the *Rickettsiella* genera, it is possible that *C. burnettii* can infect neurons. Additionally, there is evidence that neurological involvement in disease is possible. Previously, it has been reported that patients with Q fever can experience meningoencephalitis and encephalitis, both of which can cause behavioral changes similar to those of AD [19]. Some patients who experienced these symptoms had IgG and IgM antibodies specific to *C. burnettii* in serum and CSF, although it has been reported rarely [19]. In 2002, Ayres et al. performed a prospective follow-up study of patients diagnosed with Q fever in an outbreak in 1989 to assess the long-term effects of the disease [46]. Of the 85 patients who responded to questionnaires, none presented with neurological involvement, and only two of the deceased patients included dementia as the cause of death [46]. Despite its rare neurological involvement and close relation to the *Rickettsiella* genera, which includes several *Rickettsial* species that can infect neurons, it seems unlikely that *C. burnettii* is the cause of AD based on a literature review. Table 2 summarizes rickettsia and other bacteria associated, and the underlying mechanisms with AD.

#### 2.1.4. Helicobacter

The gram-negative bacterium *Helicobacter pylori* is a pathogen that usually colonizes the digestive tract, and it is highly prevalent, affecting over 50% of the world population chronically [20]. *H. pylori* is linked to several gastric diseases, including ulcers and cancer [20]. Additionally, the bacteria has been associated with diseases outside the digestive tract, such as ischemic heart disease, vascular disorders, and autoimmune disorders [21]. Although AD patients have been seen to have higher seropositivity rates for *H. pylori* antibodies, it is challenging to draw a direct link, due to the high prevalence of the bacteria among the global population [21]. Therefore, several studies have evaluated the possible association between *H. pylori* and AD further. When testing the cerebrospinal fluid of patients with AD, the concentration of anti-*H. pylori*-specific IgG antibodies was higher in patients with AD than those without AD, and the concentration of antibodies was correlated with the severity of infection seen in individuals with AD versus individuals without AD [21]. Wang et al. used a mouse model to observe if *H. pylori* influenced learning and memory and saw that following intraperitoneal injection of the bacterium, mice showed impaired spatial learning and memory [22]. It was also seen that *H. pylori* infection was not associated with neuron loss, but rather the hippocampus and cortex of rats infected with the bacteria had increased levels of Aβ_40_ and Aβ_42_, the latter of which caused more memory impairment [22]. Several synaptic receptors on the dendritic spines of the rat hippocampi were impaired, potentially due to *H. pylori* affecting synaptic plasticity [22].

When β-secretase and γ-secretase cleave amyloid precursor protein, Aβ is formed, potentially leading to the Aβ plaques seen in AD [22]. One component of γ-secretase, PS-2, was seen in higher quantities in the hippocampi and cortexes of mouse brains that were infected with *H. pylori* filtrate, and Aβ_42_ production and PS-2 expression were increased in vitro when cells that continuously overexpress amyloid precursor protein were incubated with *H. pylori* filtrate [22]. Together, these results indicate that upregulated γ-secretase activity induced by *H. pylori* surface proteins could increase Aβ_42_ production [22]. This is one proposed mechanism of how *H. pylori* may affect the development of AD by stimulating the formation of Aβ plaques.

Hyperphosphorylation of tau forming neurofibrillary tangles is another marker of AD, and *H. pylori* may also increase the production of this characteristic. In mice infected with *H. pylori*, tau hyperphosphorylation was higher than that of mice infected with an *E. coli* control, and prolonged infection lead to sustained hyperphosphorylation [23]. It was also seen that tau hyperphosphorylation was located in the cytoplasm of hippocampal neural fibers, a brain location affected in AD [23]. When neuroblastoma cells were incubated with *H. pylori* filtrate, tau hyperphosphorylation was increased in a time-dependent manner, suggesting that prolonged exposure to *H. pylori* could cause this pathology in AD brains [23]. The phosphorylation of tau is mediated by several enzymes, including glycogen synthase kinase-3β (GSK-3β). When measuring the enzyme activity in cells treated with *H. pylori* filtrate, GSK-3β seems to be activated through a series of phosphorylation, and this observation was confirmed when GSK-3β was inhibited, leading to the rescue of hyperphosphorylation of tau [23]. Taken together, these results propose mechanisms by which *H. pylori* induces hyperphosphorylation of tau and expression of Aβ_42_ and provide evidence that the bacteria may cause the pathology seen in AD.

#### 2.1.5. Oral Bacteria

Evidence is increasing that periodontal disease, and the bacteria associated with the disease, might contribute to the pathology of AD, suggesting a few bacteria that are potential causes. As a chronic disease that affects mainly older populations, periodontal disease is an infection of the gums that can lead to tooth damage, tooth decay and loss, and jawbone decay. As individuals with AD and periodontal disease age, it is seen that as periodontal disease worsens, increased cognitive decline is observed, suggesting a correlation [24]. It is also hypothesized that the oral microbiome may play a role in AD as well. The composition of human microbiota is a subject of great interest recently, as there is evidence that the bacterial composition can influence health, the immune system, and disease. As humans age, the microbiota of various parts of the body shifts, including the oral microbiota [49]. For example, *Actinomyces* species are more prevalent in older populations, whereas there are very few differences in the oral microbiota composition of age groups under 60 years [49]. It is hypothesized that inflammation seen in periodontal disease could lead to the brain inflammation observed in AD. The innate immune response to bacterial components, such as lipopolysaccharide, flagellin, and other pathogen-associated molecular patterns (PAMPs), trigger the release of pro-inflammatory cytokines [25]. For example, several pro-inflammatory cytokines and Tumor Necrosis Factor-α (TNF-α) are elevated in AD patients with periodontal disease [24]. Inflammation has a known association with AD, and it is hypothesized that various bacteria involved in periodontal disease may contribute to inflammation and, therefore, AD [24]. In a study that evaluated the levels of TNF-α in AD patients compared to normal patients, TNF-α was significantly higher in AD patients [50]. However, it is still unknown whether periodontal bacteria are directly causing inflammation in the brains of individuals with AD.

IgG antibodies to several bacterial species have been observed in AD patients in higher quantities than in normal patients, such as *Aggregatibacter actinomycetemcomitans*, *Tannerella forsythia*, and *Porphyromonas gingivalis* [50]. Kramer et al. compared IgG antibodies to these bacteria in the serum of AD patients and normal patients and saw antibodies to at least one of the tested bacteria present in about 72% of AD patients, but only 38% of normal patients [50]. These results indicate that bacteria associated with periodontal disease may influence AD. Of particular interest is *Porphyromonas gingivalis*, which has been observed to increase in the oral microbiome as individuals age, especially in those with periodontitis [49]. *P. gingivalis* possesses several virulence factors, such as lipopolysaccharide (LPS), that stimulates a robust inflammatory response, which may cause some of the inflammation seen in AD brains [25]. Because the bacteria have been shown to activate the complement pathway, in addition to being detectable in the brain, it is suggested that *P. gingivalis* infection in patients with chronic periodontitis could lead to AD development and pathology [26]. There is increasing evidence that *P. gingivalis* infection is associated with cognitive decline. It is known that *P. gingivalis* releases cysteine proteases called gingipains, which aid the bacteria in evading the host immune response, colonizing affected areas, and acquiring nutrients [26]. Because gingipains can stimulate a robust host immune response, Dominy et al. hypothesized that gingipains could play a role in AD pathogenesis [26]. Two gingipains, RgpB and Kgp, were seen in higher concentrations in AD brains compared to normal brains, and both were associated with higher tau load and ubiquitin, a protein associated with Aβ plaques [26]. RgpB was localized in neurons, astrocytes, tau tangles, and Aβ plaques in AD brains, indicating that gingipain is associated with AD pathology [26]. PCR analysis revealed that *P. gingivalis* DNA was present in the cerebral cortexes and CSF of AD brains and normal brains that were positive for Kgp, providing evidence that the bacterium can travel from the oral cavity to the brain and CSF [26]. To provide a further association between *P. gingivalis* and AD pathology, mice were infected orally with the bacterium, and it was seen that *P. gingivalis* could enter the brain and induce the formation of Aβ plaques [26]. Taken together, these results indicate that *P. gingivalis* infection in patients with chronic periodontitis may contribute to AD development and pathology, potentially due to the production of gingipains. Some organisms use biofilms as a means of adhering to surfaces and colonizing an area of the body to cause infections and diseases. As such, biofilms are a crucial virulence factor for certain bacteria to persist in areas of the body where there is high movement, such as the urinary tract and the oral cavity. Diseases such as pneumonia, urinary tract infections, cardiovascular diseases, and joint infections of knee can be mediated by oral bacteria, including *Streptococcus pneumoniae* and *Pseudomonas aeruginosa* [47,48]. It is hypothesized that certain oral bacteria can enter the bloodstream through breaks in the oral cavity resulting from periodontal disease, as seen in elderly individuals who develop extra-oral infections with oral bacteria. Therefore, there is reason to believe that oral bacteria, like the aforementioned *P. gingivalis,* can enter the bloodstream and travel to the brain to cause infection, increased release of pro-inflammatory cytokines, and inflammation, which may lead to AD [47,51,52]. Other periodontal bacteria that form biofilms, such as *Actinomyces naeslundii*, *Prevotella intermedia*, *Fusobacterium nucleatum*, and *Treponema denticola*, have been seen to be associated with AD development and progression based on serum antibodies detected in AD patients [47].

## 3. Spirochetes as Causative Agents of AD

Most notably, spirochetes are bacteria that can also form biofilms, and there is increasing evidence regarding infections with specific species. *Borrelia burgdorferi*, and several species of treponemes are some of the most studied spirochetes regarding AD. As these bacteria also form biofilms, it is thought that there may be a mechanism through which *Borrelia burgdorferi* and *Treponema pallidum* can induce AD pathology [47,53,54]. Spirochetes are gram-negative, spiral-shaped bacteria that use endoflagella to exhibit high motility in vitro and in vivo. When exploring the hypothesis that spirochete infections could contribute to AD pathogenesis, non-specific spirochetes were detected in senile plaques based on peptidoglycan immunoreactivity [27]. Spirochetes were visualized in the neurofibrillary tangles and senile plaques in the brains of individuals with AD, and peptidoglycan was also located near Aβ deposits [27]. Several studies show combined evidence that spirochetes are detectable in the brains of about 90% of AD patients, whereas they were often not present in non-AD brains, with periodontal spirochete presence being even higher at over 93%, showing a significant association of spirochetes with AD [27]. In the interaction of spirochetes and the human immune system, toll-like receptors (TLRs) on innate immune cells recognize components of spirochetes, such as lipopolysaccharide (LPS) and peptidoglycan [27]. Infections with spirochetes induce cytokine release, and spirochete lipoproteins, including lipopolysaccharide-binding protein (LBP), can activate TLR to stimulate TNF production [30]. TNF production and cytokine release induce inflammation locally and systemically in infected individuals, but it is unclear whether or not the inflammation seen in AD brains is due to spirochete infections. Therefore, evidence is needed to determine if there is an association between AD and spirochetes. After these early results, researchers hypothesized that two of the potential spirochetes could be *Treponema pallidum* or *Borrelia burgdorferi*, based on the neurological symptoms that the bacteria are known to cause, and these organisms have been highly studied in relation to AD.

### 3.1. Borrelia

Lyme disease is a tick-borne illness that results when a tick carrying several species of bacteria, most notably the spirochete *Borrelia burgdorferi*, takes a bloodmeal from a human. The bacteria enter the human, and it is known to disseminate throughout the body to cause the pathological and clinical symptoms of Lyme disease. Although Lyme disease can be treated with antimicrobials when recognized before dissemination, when left untreated, the infection can spread to other organs, including the brain, where it can cause the patient to exhibit neurological symptoms [55]. Late Lyme disease is characterized by inflammatory arthritis, resulting from pro-inflammatory factors, such as TNF-α [55]. Additionally, *B. burgdorferi* has been associated with the development of psychiatric disorders, including depression, bipolar disorder, and anxiety disorders [56]. Due to the inflammation seen in late Lyme disease, neurological symptom presentation, and the spirochete’s ability to disseminate to other organs, it has been suspected that *Borrelia burgdorferi* infections may be involved in AD.

Lyme neuroborreliosis (LNB) is caused by *B. burgdorferi* and can result in meningoencephalomyelitis and altered motor functions associated with the peripheral nervous system [57]. Early studies indicated that antigens specific for *B. burgdorferi* are detectable in the brains of AD patients with Lyme neuroborreliosis, and bacterial DNA based on PCR analysis was also present [27]. One of the hypotheses linking AD to *B. burgdorferi* is that infection with the bacterium could induce Aβ deposition in the brain. When neurons, astrocytes, and mammalian microglial cells are infected with *B. burgdorferi*, Aβ precursor protein and Aβ plaque aggregation and deposition are observed to increase with time, demonstrating that the bacterium could induce this pathological feature of AD as Lyme disease progresses [58]. It is also thought that AβPP is the cause of the Aβ plaque formation due to *B. burgdorferi* infection, and higher levels of AβPP were seen in Borrelia-infected cells [58]. Therefore, it can be assumed that infection with *B. burgdorferi* induces the Aβ plaque deposition in neuronal cells through increased AβPP. Hyperphosphorylation of tau is also seen in these infected cells, further suggesting that *B. burgdorferi* infection may induce AD pathology [30]. Evidence has indicated that one of the components of *B. burgdorferi*, lipopolysaccharide (LPS), could have a role in causing AD pathology, specifically tau hyperphosphorylation [58]. When cells were exposed to LPS, AβPP levels increased as time increased, coinciding with an increase in tau hyperphosphorylation [30]. This suggests that the LPS component of *B. burgdorferi* could be the cause of AD pathology. The observation that Lyme disease is often a co-infection with several other pathogens— such as the previously described *C. pneumoniae*, HSV-1, and *H. pylori*—further suggests that *B. burgdorferi* might act similarly to these pathogens in reference to AD development, or that their co-infection contributes to AD pathology [27].

While studies have shown an association between AD pathology and *B. burgdorferi*, exactly how the bacterium can induce it is unclear. One hypothesis is that biofilm formation creates the Aβ plaques characteristic of AD. This hypothesis stems from the observation that *B. burgdorferi* forms biofilms in vitro and in the joints of individuals with Lyme disease, an area affected by the disease [59]. Studies have also shown that Aβ plaques are composed of biofilms and their constituents, and spirochetes can express AβPP, suggesting that spirochetes themselves could induce the plaque formation characteristic of AD [59]. In an in vitro culture, it was confirmed that *B. burgdorferi* forms biofilms and aggregations that increase over time, and these biofilms contained Aβ and AβPP when analyzed with immunostaining [59]. When examining senile plaques harvested from the brains of patients diagnosed with AD, *B. burgdorferi* peptidoglycan-specific antigens, *B. burgdorferi*-specific antigens, and *B. burgdorferi* DNA were discovered [59]. It was also seen that these biofilms react to anti-AβPP and anti-Aβ antibodies [59]. These results indicate that biofilms formed by *B. burgdorferi* exhibit similar pathology to one of the hallmarks of AD, Aβ plaque deposition. Upon direct examination of cortical sections of AD patients, biofilms formed by *B. burgdorferi* were present in the brain samples, as shown by immunoreaction of anti-*B. burgdorferi* antibodies and anti-Aβ antibodies [59]. Overall, these observations indicate that senile plaques contain bacterial amyloid, potentially due to biofilm formation by the spirochete *B. burgdorferi*. Therefore, it can be hypothesized that *B. burgdorferi* infection may cause AD pathology and induce AD symptoms.

### 3.2. Treponema

For many years, there has been evidence that treponemes are present in the oral cavity. Given previously described observations that oral bacteria in chronic periodontitis can travel to and infect the brain, it was hypothesized that *Treponema* spp. can do the same. When examining the possibility that oral treponema species can travel to the brain and induce AD pathology, Riviere et al. demonstrated that AD brains were positive for at least one treponeme species, and some AD patients had multiple species present [60]. It is noted that control subjects without AD were negative for most *Treponema* species in the brain samples, but the presence of treponemes in the oral cavities of control and AD patients did not differ significantly [60]. Therefore, it is suggested that several oral treponemes may invade the central nervous system and lead to AD pathology, although the mechanism remains unclear.

It is now known that treponemes, specifically *T. pallidum*, can invade the brain by way of penetration of endothelial cell tight junctions, presenting evidence that infection with *T. pallidum* can cause disseminated infections in other areas of the body, including the brain [61]. The spirochete *Treponema pallidum* is the causative agent of syphilis, a progressive sexually transmitted infection that can infect the brain when left untreated, resulting in neurosyphilis. When the disease reaches this stage, the bacterium has been known to induce dementia with a persistent brain infection, potentially decades after primary infection [31]. For many years, *T. pallidum* and its effects on the brain have been studied. It is known that the spirochete can persist in the brain and lead to general paresis, characterized by brain inflammation and muscular weakness, and early studies indicated that spirochetes gathered in the cortexes and neuronal cells of patients with neurosyphilis [31]. Additional early evidence described neurofibrillary tangles in patients with neurosyphilis, leading to the hypothesis that there is a relationship between *T. pallidum* and AD. Via direct examination of the brain, spirochetes, specifically *T. pallidum*, can form masses or aggregates in the brain, which highly resemble Aβ senile plaques in individuals with AD [31]. Upon central nervous system invasion, *T. pallidum* can cause inflammation and amyloid plaque formation [50]. It is noted that the Aβ plaques formed by *T. pallidum* highly resemble the Aβ plaques observed in AD, indicating that spirochetes, specifically *T. pallidum*, may contribute to AD development [31]. However, several cases have been presented that demonstrate Alzheimer’s dementia can be mimicked by neurosyphilis, and neurosyphilis may be mistaken for early-onset AD. For example, in 2012, a Bulgarian man presented with signs typical of early AD, including memory loss [62]. When scored with the Mini-Mental State Examination (MMSE) cognitive functioning scale, the man presented with low ability to retain new information, verbal impairment, and disorientation [62]. These clinical symptoms closely mimic some of the cognitive impairments seen with AD, but hemagglutination assays showed a positive result for *T. pallidum* [62]. In a similar case, a 40-year-old man presented with cognitive decline and behavioral changes, prompting clinicians to suspect AD [63]. An MRI showed significant atrophy of the medial temporal lobe, again signaling AD [63]. However, hemagglutination assay performed on the patient’s CSF indicated the presence of *T. pallidum* [63]. These presented cases prompted further examination after the initial diagnosis of early-onset AD because of the patients’ ages. These cases beg the question if older patients diagnosed with AD may be misdiagnosed with the disease when they have neurosyphilis. Given the findings that *T. pallidum* may induce AD pathology, and the presented cases, it might be of interest to examine if patients diagnosed with AD by the presence of Aβ plaques and NFTs are positive for *T. pallidum* with a hemagglutination assay.

## 4. Evidence for Treponeme as the Causative Agents of AD

The preceding review has summarized the potential pathogenic causes of AD. *Candida albicans* and *Toxoplasma gondii* have both been detected in the brains of AD patients. Several viruses, including HSV-1 and CMV, have also shown an association with AD. Bacteria, such as *C. pneumoniae*, *H. pylori*, and *P. gingivalis* were also described. As spirochetes have been widely studied, *B. burgdorferi* and *T. pallidum* were examined in depth in this review. With the information compiled, it is decided that treponemes, specifically oral treponemes, should be studied further.

As previously stated, several bacterial species that contribute to periodontal disease are thought to be associated with AD due to the apparent ability of these bacteria to travel to the brain through means of the oral cavity. Given that spirochetes have a noticeable correlation with AD and some periodontal bacteria are spirochetes, studies have examined the possibility that oral spirochetes, specifically those that are considered pathogenic, could be linked to AD. For example, the spirochete *Treponema denticola* has been seen in both adults and children with periodontal disease, although it is more commonly present in older individuals [64]. As mentioned previously, periodontitis is a progressive oral infection of the gums that can eventually lead to tooth decay and tooth loss. Although there is sufficient evidence of *T. denticola* in periodontal disease, evidence of the pathogen’s ability to disseminate beyond the oral cavity is accumulating. Foschi et al. demonstrated the effects of *T. denticola* alone and in combination with other periodontal pathogens, *Tannerella forsythia* and *Porphyromonas gingivalis*, in severe combined immunodeficiency (SCID) in wild-type mice [65]. SCID mice more frequently developed oral abscesses than all other groups when infected with *T. denticola* only, suggesting that more severe periodontal infections may be caused by this pathogen [65]. When evaluated for splenomegaly, it was seen that all experimental groups developed splenomegaly with similar incidence and severity, indicating that *T. denticola*, as well as other oral bacteria, can disseminate to organs beyond the oral cavity [65]. *T. denticola* was also detected in distant organs, including the spleen, heart, and brain of 4/10 SCID mice, with *T. denticola* being more frequently seen in the heart and spleen [65]. Only 1/10 SCID mice had *T. denticola* present in the brain [65]. Although it was not common in the brain, these results indicate that *T. denticola* can disseminate beyond the oral cavity and infect other sites.

Because of previous evidence that *Borrelia burgdorferi* forms biofilms that may contribute to AD pathology, it is possible that the same could be true for *T. denticola*, as they are both spirochetes. *T. denticola* has been seen to form biofilms in the oral cavity in conjunction with the aforementioned *P. gingivalis* [66,67]. Grenier demonstrated that when *T. denticola* and *P. gingivalis* are inoculated in brain heart infusion broth with vitamin K, the combination grew better than either alone [68]. In an in vitro experiment, Yamada et al. showed that *T. denticola* and *P. gingivalis* were able to more effectively colonize when incubated together than *T. denticola* with two other periodontal bacteria, *F. nucleatum* and *T. forsythia*, and the biofilm formed by *T. denticola* and *P. gingivalis* showed greater adherence [67]. Seeing as *P. gingivalis* has been previously found in the brains of AD patients and the bacteria can induce Aβ plaques, it is possible that the biofilms formed synergistically by these two bacteria could have a role in AD pathology.

The evidence previously described demonstrates that spirochetes may contribute to AD pathology, and *T. denticola* shows similar potential. It is possible that several treponemes might be associated with AD. Riviere et al. used various methods to observe if any oral treponemes were present in AD brains [60]. In samples from frontal lobe cortexes, at least one *Treponema* species was observed in 14/16 AD brains, while only 4/18 control cortexes were positive for any *Treponema* species as seen by PCR [60]. The *Treponema* species observed in AD brains were *T. amylovorum*, *T. denticola*, *T. maltophilum*, *T. pectinovorum*, and *T. socranskii* [60]. Additionally, some AD patients were positive for more than one treponeme [60]. In the terminal ganglia of AD brains, all subjects were positive by PCR for at least one *Treponema* species, although only three AD samples were tested [60]. Interestingly, when the saliva of living AD patients and controls were tested for treponemes, no significant differences in the species or quantity of spirochetes were detected [60]. Because there was virtually no difference in treponemes in saliva, but several *Treponema* species were present in AD brain samples more frequently than in non-AD brains, it is possible that the migration of treponemes from the oral cavity to the brain could lead to the development of AD. However, the relationship between oral Treponemes and AD pathology has yet to be further elicited.

## 5. Conclusions

Alzheimer’s disease is one of the leading causes of dementia worldwide. Although we know much about the hallmark characteristics, the exact cause of disease development remains unclear. This review aimed to evaluate and summarize the pathogens that have shown an association with Alzheimer’s disease and select a spirochete that should be studied further in laboratories. Based on scientific literature analyses, it is determined that oral treponemes fit the criteria necessary for us to proceed. Pertinent upon the observations that several treponemes can disseminate beyond the oral cavity and into the brain, there is sufficient evidence to speculate about their involvement with AD development. While *Treponema denticola* is emphasized, other oral treponemes commonly present in the oral microenvironment should also be considered. As a continuation of scientific hypotheses, a next logical step with this research could be to investigate brain tissue samples from AD patients to correlate spirochetes and other pathogens’ association with Alzheimer’s disease from an etio-pathological viewpoint.

## Figures and Tables

**Table 1:** A summary of fungi, parasites, and viruses that have been studied in association with AD, the characteristics of AD that are concurrent with the organism, and the hypothesized mechanism of causation.

Organism with Potential AD Correlation	Evidence of Organism in Brain	AD Characteristics Associated with Organism	Hypothesized Mechanism of Causation
*Candida albicans* (and other fungal species)	Fungal microtubules present in brain tissue and cytoplasm of neurons [4]; DNA and polysaccharides present in AD brain tissue [3]	Tau increased in neurons with fungal structures [5]; Aβ formed in blood vessels with fungal hyphae present [5]	Fungi increases tau hyperphosphorylation to lead to NFTs; Aβ plaques can form in response to fungi
*Toxoplasma gondii*	Cysts formed in CNS and brain [6]; Alteration of NMDAR pathway and function [6]	Parasite induces Aβ plaques and colocalization of parasitic cysts [6]; Tau hyperphosphorylation induced with infection [6]; Loss of olfaction and decreased synaptic transmission [6]	Cysts induce Aβ and tau hyperphosphorylation [6]; NMDAR receptor is altered, leading to AD pathophysiology [6]
Herpes Simplex Virus Type 1 (HSV-1)	Viral DNA present after infection clears [8]	Viral encephalitis leads to cognitive decline [7]; Viral DNA colocalized with Aβ plaques [8]; Tau hyperphosphorylation increased in infected cells [9]	APOE-ε4 gene may be a risk factor [33]; Virus phosphorylated tau at T212 and S214 [9]
Human cytomegalovirus (CMV)	Virus present in latent infections [10]	Aβ plaques induced by CMV in human cells [10]; Seropositive samples correlate with increased IFN-γ and NFT load [10]	Reactivation increases IFN-γ and TNF-α secretion to cause Aβ plaques [10]

**Table 2:** A summary of bacteria that have been studied in association with AD, the characteristics of AD that are concurrent with the bacterium, and the hypothesized mechanism of causation.

Bacterium with Potential AD Correlation	Evidence of Bacterium in Brain	AD Characteristics Associated with Bacterium	Hypothesized Mechanism of Causation
*Chlamydophila pneumoniae*	PCR detection in hippocampus and temporal lobes of AD brain tissue [11,12,13]	Aβ plaque colocalization [12]	Inflammation due to infected microglia inducing Aβ plaque formation [11,13]
*Rickettsia rickettsii*	Infected rat cerebellar granular neurons undergo apoptosis [16]	Neuronal death via apoptosis [16]	
*Helicobacter pylori*	Increased specific IgG higher in AD patients [20]	Higher levels of Aβ in rat brain tissue [21]; Increased tau hyperphosphorylation [22]	
*Porphyromonas gingivalis*	Specific IgG antibodies detected in brain [44]; PCR detection in cerebral cortexes [25]; Gingipains seen in higher concentration in AD brains [25]	Gingipains associated with higher tau load and ubiquitin [25]	Immune response to virulence factors leads to brain inflammation [25]
*Borrelia burgdorferi*	PCR detection of DNA in brain [26]	AβPP increased and Aβ plaque formation induced when cells are infected [47]; Tau hyperphosphorylation in infected cells [29,47]	LPS causes tau hyperphosphorylation [47]; Biofilms create Aβ plaques [48]
*Treponema pallidum*	Direct observation of bacterium in brain tissue cells [30]	NFTs seen in patients with syphilis [30]; Bacterium forms aggregates that resemble Aβ plaques [30]	Inflammation induces Aβ plaque formation [30]

## Data Availability

No additional data: all data is submitted in this review.
